# Distinguishing Alzheimer’s Disease Patients and Biochemical Phenotype Analysis Using a Novel Serum Profiling Platform: Potential Involvement of the VWF/ADAMTS13 Axis

**DOI:** 10.3390/brainsci11050583

**Published:** 2021-04-30

**Authors:** Jay S. Hanas, James R. S. Hocker, Christian A. Vannarath, Megan R. Lerner, Scott G. Blair, Stan A. Lightfoot, Rushie J. Hanas, James R. Couch, Linda A. Hershey

**Affiliations:** 1 Department of Biochemistry, University of Oklahoma Health Sciences Center, Oklahoma City, OK 73104, USA; James-Hocker@ouhsc.edu (J.R.S.H.); Christian-Vannarath@ouhsc.edu (C.A.V.); Rushie99@gmail.com (R.J.H.); 2 Department of Surgery, University of Oklahoma Health Sciences Center, Oklahoma City, OK 73104, USA; Megan-Lerner@ouhsc.edu (M.R.L.); Scott-Blair@ouhsc.edu (S.G.B.); 3 Veterans Administration Hospital, Oklahoma City, OK 73104, USA; SL20008bl@outlook.com; 4 Department of Neurology, University of Oklahoma Health Sciences Center, Oklahoma City, OK 73104, USA; James-Couch@ouhsc.edu (J.R.C.); linda-hershey@ouhsc.edu (L.A.H.)

**Keywords:** Alzheimer’s disease (AD), biochemical phenotype analysis, serum profiling, mass spectrometry, VWF/ADAMTS13 axis, traumatic brain injury (TBI), SSPO AD biomarker

## Abstract

It is important to develop minimally invasive biomarker platforms to help in the identification and monitoring of patients with Alzheimer’s disease (AD). Assisting in the understanding of biochemical mechanisms as well as identifying potential novel biomarkers and therapeutic targets would be an added benefit of such platforms. This study utilizes a simplified and novel serum profiling platform, using mass spectrometry (MS), to help distinguish AD patient groups (mild and moderate) and controls, as well as to aid in understanding of biochemical phenotypes and possible disease development. A comparison of discriminating sera mass peaks between AD patients and control individuals was performed using leave one [serum sample] out cross validation (LOOCV) combined with a novel peak classification valuation (PCV) procedure. LOOCV/PCV was able to distinguish significant sera mass peak differences between a group of mild AD patients and control individuals with a *p* value of 10^−13^. This value became non-significant (*p* = 0.09) when the same sera samples were randomly allocated between the two groups and reanalyzed by LOOCV/PCV. This is indicative of physiological group differences in the original true-pathology binary group comparison. Similarities and differences between AD patients and traumatic brain injury (TBI) patients were also discernable using this novel LOOCV/PCV platform. MS/MS peptide analysis was performed on serum mass peaks comparing mild AD patients with control individuals. Bioinformatics analysis suggested that cell pathways/biochemical phenotypes affected in AD include those involving neuronal cell death, vasculature, neurogenesis, and AD/dementia/amyloidosis. Inflammation, autoimmunity, autophagy, and blood–brain barrier pathways also appear to be relevant to AD. An impaired VWF/ADAMTS13 vasculature axis with connections to F8 (factor VIII) and LRP1 and NOTCH1 was indicated and is proposed to be important in AD development.

## 1. Introduction

Alzheimer’s disease (AD) is the most common form of dementia, affecting nearly 10% of adults 65 and over [1]. The AD brain pathologies (e.g., neuron cell loss, neurofibrillary tangles) are present in the normal aging brain but are more advanced in AD and that severity is a characteristic of the disease [1]. AD can also be defined biologically by the biomarkers associated with these brain pathologies [2]). Early identification and monitoring of AD are important aspects of elderly memory care. Magnetic resonance imaging (MRI) has shown heterogeneity during the analysis of early stages of AD, so the diagnosis and monitoring cannot always rely on neuroimaging [3,4]. Amyloid positron emission tomography (PET) and fluorodeoxyglucose (FDG)-(PET) of the brain were shown to be sensitive and specific research tools to distinguish early stages of AD from dementia with Lewy bodies (DLB), frontotemporal dementia (FTD), or normal aging [5,6,7,8]. Tau PET is also useful in AD monitoring [9].

However, these tests are often not widely available or easily affordable outside of research settings. What is needed for aiding in the identification and monitoring of early-stage AD are accurate and low-cost testing aids for individuals at high risk for these pathologies, such as traumatic brain injury (TBI) patients especially with mild cognitive impairment [10]. Additionally, gleaning underlying biochemical mechanistic understanding of AD from such analyses would be helpful for developing future biomarkers and therapeutics.

Since peripheral blood is an easily obtainable, minimally invasive tissue for disease biomarker analysis, the blood derivatives serum and plasma are ideal vehicles for biomarker analysis and understanding of early progression of AD [1]. Such studies presently range from promising to uneven. Studies of plasma amyloid β-42 levels have shown mixed results. For example, no significant differences between AD cases and controls were identified in some studies, elevated levels in other studies, while reduced levels were measured in still others [11]. Tau phosphorylated at threonine 181 (p-tau181) or 217 (p-tau 217) appears to be a better AD predictor [12,13]. While the presence of the apolipoprotein E-ε4 allele (*APOE*-*ε4*) is associated with lower levels of APOE protein in plasma [14], studies have shown inconsistent associations between reduced plasma APOE levels and the presence of AD [15]. One study used a panel of 4 plasma biomarkers to predict the progression through early-stage AD, but this panel demonstrated a sensitivity (true positive rate) of only 74% [16]. A longitudinal study of cognitive decline in older adults used a panel of 8 blood-based markers, including APOE, plasma amyloid β 42/40 ratio, telomere length, serum glucose, cystatin C, C-reactive protein (CRP), interleukin-6 (IL-6), and albumin [17]. After 11 years of follow-up, the 5 blood-based markers that correlated best with cognitive decline were APOE (present/absent), cystatin C, serum glucose, CRP, and IL-6. A more promising AD biomarker approach utilized a combination of amyloid PET analysis of the brain with mass spectrometry (MS) of plasma ratio levels of amyloid β 42/40, achieving a sensitivity of 88% [18].

A blood-based biomarker approach not yet explored in distinguishing and monitoring of early-stage AD is the use of an unfractionated all-liquid electrospray ionization (ESI) mass spectrometry (MS) serum mass peak profiling platform. This approach was successful in discriminating and biochemically monitoring other disease states of the brain including traumatic brain injury (TBI) and epilepsy [19,20,21]. While the AD studies described above have used costly and time-consuming analytical procedures, this novel ESI-MS serum mass profiling platform needs minimal sample preparation followed by injection into a robust and efficient MS instrument with software-controlled mass peak analysis.

The hypothesis of serum biomolecule profiling is that the levels and kinds of biomolecules in serum will reflect specific changes in physiology, in particular those changes accompanying specific disease states as well as the bodily systemic responses to those disease processes [19,20,21]. This ESI-MS procedure applied in this present study examines a large number of different biomolecules in sera, whereas other biomarker platforms focus on a single component or relatively small numbers of components. Examining a large number of biomolecules at the same time is important because analysis of more components increases the ability of the platform to discriminate disease states from each other and from homeostasis [19,20,21,22]. The goal of the current study is to assess to what extent serum mass peak profiling using ESI-MS is able to discriminate sera from mild or moderate AD patients as well as versus control individuals. In addition, this MS platform was utilized to analyze the serum peptide/protein differences and similarities between AD patients and controls using MS/MS structure determinations. This gene expression information was analyzed by bioinformatics software to predict possible affected cellular pathways and phenotypes in early-stage AD. Potential affected pathways include those involving apoptosis/neuronal cell death, vasculature, and AD/dementia/amyloidosis. A VWF/ADAMTS13 protein axis with inputs from LRP1 and F8 is proposed to be important in impaired cerebral vascular events in AD development.

## 2. Materials and Methods

### 2.1. Patients and Clinical Samples

Table 1 (panels I and II) exhibits study volunteer demographic, mental testing, and daily living activity information, and inclusion/exclusion criteria. Volunteer recruitment began on 6/18/13 and ended on 5/31/16. All AD patients (mild or moderate) and control individuals were community-dwelling adults, who could read and understand English. They had similar mean ages (74–77), slight group imbalances toward females, and minimal tobacco smoking present. All volunteers had a Clinical Dementia Rating (CDR) score in the range of 0–2, which includes normal, mild, or moderate AD/dementia [23]. Within this range, volunteers with a CDR between 0 and 0.5, indicating mild cognitive impairment (MCI), were excluded. All controls had a CDR of 0. Mini-Mental State Exam (MMSE) scores were in the proper ranges for controls, mild AD, or moderate AD; AD patients met criteria for the clinical diagnosis of probable AD, according to McKhann et al. [24]. Individuals were excluded from this study if they:

(a) Had a receptive or expressive aphasia;
(b) Were in a bedridden state;
(c) Were legally blind;
(d) Had severe dementia (CDR = 3.0, or MMSE < 10);
(e) Had severe depression, anxiety, or psychosis, or Neuropsychiatric Inventory (NPI) score according to Cummings et al. [25], of >5/12;
(f) Had a history of cancer;
(g) Had a diagnosis of MCI (0 < CDR ≤ 0.5);
(h) Were University of Oklahoma (OU).

Physicians staff members or employees. Patients and controls signed informed consent before they were assessed with the cognitive, functional and neuropsychiatric screening tools and before the blood samples were drawn. Serum aliquots (100 µL) were frozen at −80 °C, and not reused after initial freezing and thawing. Serum was collected from peripheral blood in accordance with biomarker guidelines as previously described [26]. This protocol was approved by the OU Physicians IRB (institutional review board) for human studies.

### 2.2. Electrospray Mass Spectrometry of Sera from Mild or Moderate AD Patients, and from Control Individuals

**Figure 1:**
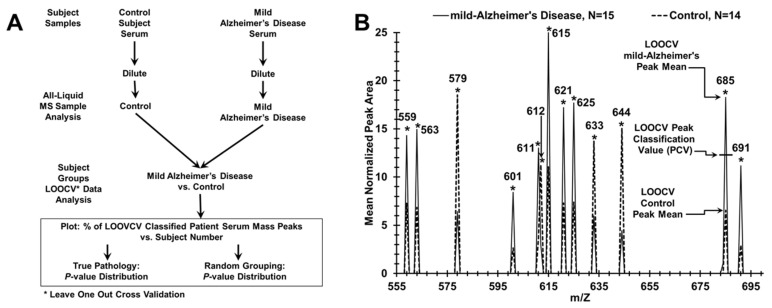
Analytical procedures involving LOOCV/PCV for distinguishing AD patient and control groups using a MS serum profiling platform. (**A**) Flow chart for serum sample handling and mass spectrometry for binary AD patient/subject group analysis. (**B**) Serum mass peak Scoring for LOOCV/PCV (leave [one serum sample] out cross validation/peak classification value) procedure to classify mass peaks as either “mild AD” or control from a “left out” sample, over a narrow range (555–695 *m*/*z* is displayed) of significant group discriminatory mass peaks. The PCV example is exhibited on peak 685 which is used to classify “left out” peaks as either “mild AD” (peak area above this PCV) or control (peak area at or below this PCV).

**Figure 2:**
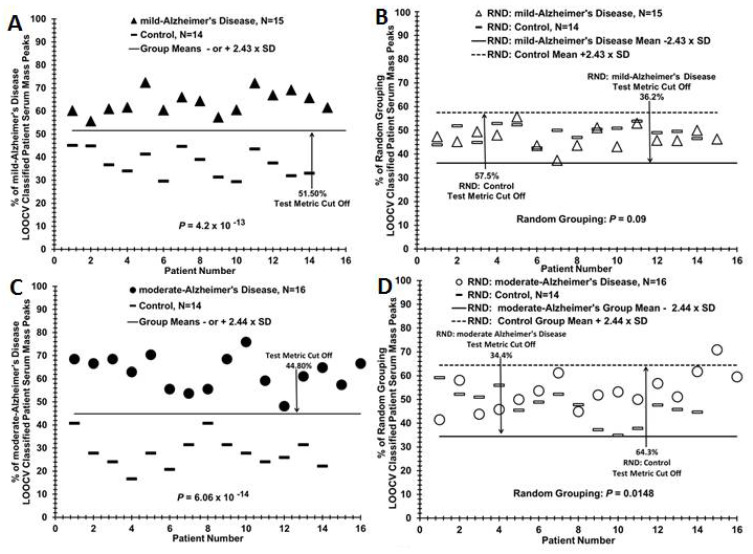
Distinguishing sera of mild or moderate AD patients from control individuals by MS serum mass profiling (**A**) Serum discrimination of mild AD patients (dark triangles) from controls (dashes) by % of LOOCV/PCV classified mass peaks. A cut off value is present (− or + SDs from the mild AD or control groups, respectively) to determine test metric values (e.g., true positives). (**B**) Non-serum sample discrimination when the two different sample groups in A are mixed together randomly followed by the same LOOCV/PCV mass peak analysis. (**C**) Serum discrimination of moderate AD patients (dark circles) from controls (dashes). (**D**) Non-serum discrimination upon randomization of sera samples between the two groups in C followed by the same LOOCV/PCV analysis.

**Figure 3:**
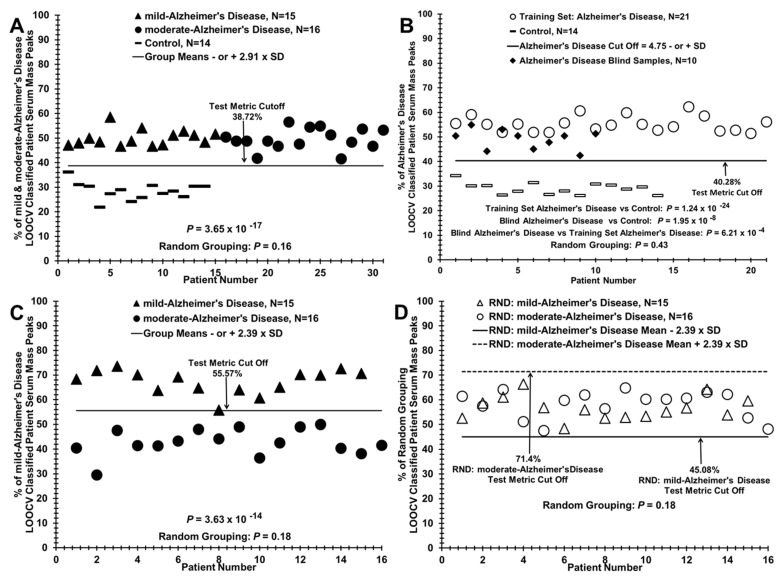
Assigning pathology grouping to a blinded set of AD patient sera vs. controls and discriminating sera of mild AD patients from moderate AD patients. (**A**) Distinguishing mild and moderate AD patient sera as a group (*N* = 31, dark triangles and circles) versus controls (*N* = 14, dashes) for formation of the training set used in panel B. (**B**) Assigning correct group pathology to a blinded “left out” group of AD patients (*N* = 10, dark diamonds) from panel A against a training set of AD patients, *N* = 21 (open circles) and controls *N* = 14 (dashes) from panel A. (**C**) Distinguishing mild AD patient sera (dark triangles) from moderate AD patient sera (dark circles) using the LOOCV/PCV procedure. (**D**) Randomization of the two groups in panel C showing non-discrimination.

**Figure 4:**
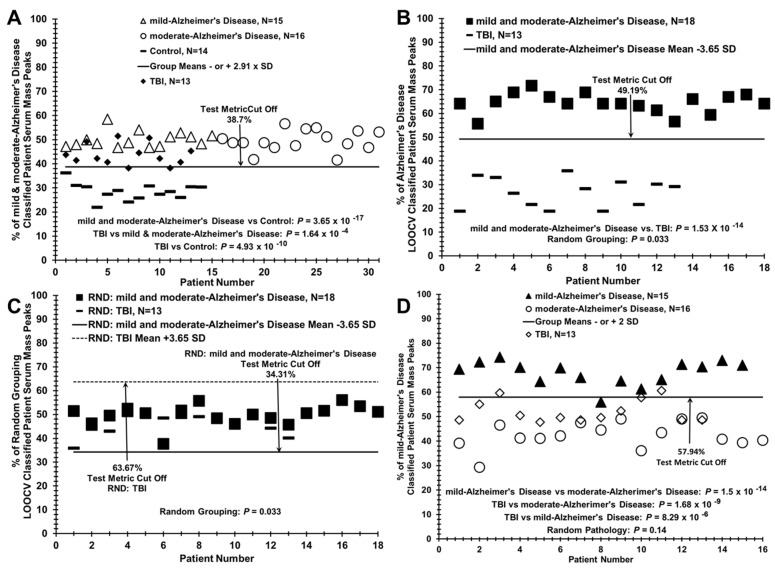
LOOCV/PCV procedure sequesters mild TBI patient sera with AD sera when compared to controls, and with moderate AD patient sera when compared to mild AD patient sera. (**A**) Sequestering of TBI patient sera (*N* = 13, dark diamonds) with mild (open triangles) and moderate (open circles) AD patient sera versus controls (dashes). (**B**) Distinguishing mild and moderate AD patient sera (dark squares) from mild TBI patient sera (dashes) using LOOCV/PCV. (**C**) Randomization of sera samples between the two groups in panel B exhibiting loss of group discrimination. (**D**) Sequestering of TBI patient sera with moderate AD sera versus mild AD sera.

The Advantage LCQ ion-trap electrospray MS instrument (ThermoFisher, Inc.) was utilized for “leave one out [serum sample] cross validation” (LOOCV) analysis of serum MS spectra (Figure 1, Figure 2, Figure 3 and Figure 4) and for tandem MS/MS peptide/protein structural identifications 2for the AD patient and control sera samples listed in Tables 3 and 4. Calibration of the LCQ was performed following recommended manufacturer protocols. HPLC grade solvents were purchased from ThermoFisher. The MS serum injection sample for ionization in the MS instrument is diluted into an organic phase by adding 8 µL of serum to 2.4 mL of solution (50% methanol, 2% formic acid). The sample is then centrifuged at 14,000 rpm (16,873× *g*) for 5 min to remove precipitated higher molecular weight proteins, and aliquoted for the MS spectrum analysis.

The samples were infused by loop injection (20 µL) into the nano-source of the mass spectrometer fitted with a 20 µm inner diameter (100 µm outer diameter) fused silica (Polymicro Technologies) tip. Solvent flow was 0.5 µL/min using an Eldex MicroPro series 1000 pumping system. All instrument settings were described previously [27]. Patient sera were analyzed randomly through acquisition of high-resolution triplicate mass spectra. The spectra were sampled at a *m*/*z* (mass divided by charge) resolution of two hundredths over a *m*/*z* range of 400–2000, and positive ion spectra were averaged over a period of 20 min for each injection. Each patient’s spectral data were extracted using the manufacturer’s software (Qual Browser: version 1.4SR1) as “Nominal Mass Spectra” (whole unit intensity spectral data). MS spectral peak assignments and areas were calculated as centroid *m*/*z* peak area values (valley to valley) using Mariner Data Explorer 4.0.0.1 software (Applied BioSystems, Foster City, CA, USA). Centroid area is defined as the area of the peak calculated from its geometric *m*/*z* center.

An Expression CMS (Advion, Inc., Ithaca, NY, USA) single-quadrupole desktop ESI-MS instrument was also utilized as a MS acquisition instrument for the described serum peak classification analysis. This instrument has a mass analyzer of differing physics from the LCQ ion-trap, possesses lower resolution, and provides a lower-cost alternative “check” on the novel methodology for the disease serum sample discriminations presented here. Solvent flow of 0.23 µL/min was provided by a Harvard Apparatus Pump 11 Elite equipped with a Hamilton 250 µL gastight syringe. There was no gas flow provided. The capillary temperature was 200 °C and the voltage settings were capillary 150 V, source offset = 20 V; source span = 0.0 V; ESI = 1850 V. General modifications made to the standard ADVION system and set up included Advion Data Express version 3.3.5.2. The tip was identical to the tip used in the LCQ Advantage except the voltage was supplied through a M-572 IDEX-Health and Science conductive MicroUnion Assembly. All solvents were HPLC grade, purchased from ThermoFisher (Waltham, MA, USA). All acquisitions and calibrations were performed with the same voltages and flow rate as described for the LCQ serum analysis, but applied at a 0.23 µL/min. Expression CMS analysis of samples was conducted using 15 min averaged mass spectra, extracted for each of 3 injections for each patient sample. The remainder of spectrum processing is identical to that of the LCQ instrument.

Spectral data from both instruments were exported using a format providing rounded unit *m*/*z* and intensity values. Data were locally scaled (normalized) to a sum value of 100 intensity in non-overlapping segments of 25 *m*/*z* from 400 to 2000. MS spectral peak area assignments were calculated as centroid *m*/*z* peak area values (valley to valley) using Mariner Data Explorer 4.0.0.1 software (Applied BioSystems, Foster City, CA, USA). Centroid area is defined as the area of the peak calculated from its geometric *m*/*z* center. *m*/*z* peak area data were exported into Excel, and triplicate peak areas at each *m*/*z* value were averaged for each serum sample. To obtain information on peptide/protein changes taking place among AD patients versus control individuals, tandem MS/MS mass peak peptide/protein structure identifications were performed with the Advantage LCQ ion-trap instrument in similar fashion as described previously [19,27]. 108 unit-Dalton *m*/*z* ions encompassing the *m*/*z* range of 900–1008 were analyzed for eight AD patient (mild) sera samples and eight control individual sera samples. This particular range represents a median of approximately 100 *m*/*z* units in the 700–1200 range that previously provided serum MS/MS peptide identification data for other disease states [19,20,21].

Each parent ion at unit *m*/*z* values in the range of 900–1008 was isolated, fragmented with a 35% ionization energy setting, and MS/MS data collected for 5 min. With respect to instrument and serum stability, samples on average contained 1–2 parent ions with significant differences in standard MS spectral data between the pre- and post-MS/MS scans (5 min each) of the 108 parental ions analyzed. The diluted serum sample was renewed after each 18 peak group analysis, giving a total run time for the 18 peaks of 100 min. Analysis of MS/MS signals was performed using ThermoFisher Proteome Discoverer 1.0 (ThermoFisher, Waltham, MA, USA) sp1 on human and T. solium non-redundant databases downloaded from National Center for Biotechnology Information (NCBI), 01 February 2016 MS/MS search-related settings: [enzyme name = no-enzyme (no digest)], precursor mass tolerance =1.8 Da, fragment mass tolerance = 0.8 Da, b and y ions were scored, and dynamic modifications were noted for oxidation (C, M amino acids), phosphorylation (S, T, Y), methylation (C), all with a maximum of 4 modifications per peptide. Peptide/protein identifications required a minimum of 2 unique peptides and a cross correlation coefficient range (Xcorr) of 1.7–2.0, in line with previous studies [19]. Identified sequences were searched using Basic Local Alignment Search Tool (BLAST) against the NCBI human and non-human T. solium databases to retrieve current gene notation for analysis. A “hit” in the database search is scored for a MS/MS scan when the Xcorr, identifying a peptide sequence, is higher than the minimum cut off. Multiple scans identifying the same peptide- or protein-related sequence would be identified as multiple “hits” for that protein. For Ingenuity Pathway Analysis (IPA, QIAGEN, Hilden, Germany) to predict cellular/biochemical pathways changing in disease state comparisons, identified gene names and the number of identified MS/MS sequence “hits” (gene expression values) were imported each as log2 ratios of AD/control individual values [28]. Proteins identified by MS/MS were also manually inspected for protein function using PubMed/Medline.

### 2.3. Statistical and Quantitative Analysis

LOOCV was used to distinguish serum samples between binary groupings, for example, between mild AD patients and control individuals (sera sample flow chart, Figure 1A). LOOCV is one procedure to help reduce over-fitting (false positive generation) of large datasets [29,30,31]. The triplicate averaged serum spectra mass peak areas between groups were analyzed for significant differences at individual *m*/*z* values using Student’s t-tests (one-tailed, unequal variance, significance designated at *p* < 0.05). All significant peaks utilized for these separations were at least 0.3% of the normalized maximum peak area. For the LOOCV process, a different sample (e.g., mild AD or control) is “left out” in succession to build each unique N-1 LOOCV “left in” significant mass peak dataset. The mass peaks of each “left out” sample are then compared, peak area to peak area, to all the “left in” mass peaks in their unique N-1 LOOCV dataset. This comparison involves the use of a peak classification valuation (PCV) metric at each significant “left in” peak of the LOOCV dataset (Figure 1B). Whether a “left out” peak area falls above or below this midpoint PCV metric determines its classification. For example, in Figure 1B, peak 685 is classified as a “mild AD” peak in the “left in” database. If the 685 peak from a “left out” sample has a peak area above this PCV, then it is classified as a “mild AD”. If it falls below or equal to this PCV, then the “left out” peak is classified as “Control”. Such peak classifications are performed for all “left out” peaks in all “left out” serum samples against their respective N-1 “left in” LOOCV mass peak databases. This procedure can result in patient sera having less than 100% peak adherence to one group, resulting in serum having both a percentage of “AD peaks” and “Control peaks”. These % of total mass peaks classified (e.g., as mild AD) for the left-in dataset is assigned each “left out” sample and plotted on the y axis vs. the individual serum samples on the x-axis (e.g., in Figure 2A). To obtain potential statistical powers for group sample sizes (ability to detect type II errors–false negatives), Cohen’s d effect size values are calculated from the binary group % LOOCV means and standard deviations (SD) in Table 2 [32]. Statistical power using given sample sizes and Cohen’s d values is calculated as described [33].

This LOOCV/PCV serum mass peak analytical approach distinguishing AD patients from controls (e.g., in Figure 2A) can also analyze additional patient samples or pathological groups to assess to what extent these “new” subjects are classified into an existing two-group discriminatory binary comparison. For example, to identify how samples from a “blind set” of 10 AD patients partition between a binary comparison of related patient groups, a LOOCV training set is composed of all the moderate and mild AD samples not “left out” in the blind set, and the “left out” blind samples (mixture of mild and moderate) are then assessed against the “left in” training set (Figure 3B) using the PCV process described above. Additionally, a group of additional samples not related, or of unknown relationship, to a binary comparison (e.g., serum samples from traumatic brain injury (TBI) patients, Figure 4A) can be assessed for relatedness and differences to a particular binary comparison (moderate or mild AD vs. controls). This was performed for the raw mass peak data from sera samples from 13 male TBI patients (United States Military Veterans, previously published study [19]) who suffered a mild (loss of consciousness 1–30 min) TBI age mean = 39.9) with no signs of dementia. This is accomplished by assessing and classifying their individual raw sample mass peak areas against the PCVs. comprising that entire binary group comparison listing (e.g., mild AD vs. control) of significantly differing mass peaks. Serum sample randomizations of all group binary comparisons were performed to check for “over-fitting” of the LOOCV/PCV binary group discriminations [29]. Such random groupings of serum samples from subject groups being compared in binary fashion were obtained using the RAND (randomization) function in Excel, and manually balanced to retain gender and age ratios of the initial groups. Upon randomization, the identical mass peak LOOCV analysis was performed as described above (e.g., Figure 2B). For this process, all samples are randomized (Figure 2B) using the RAND function into both of the two study groups being compared while maintaining the original number of subjects, gender, and age in each group, creating a randomized database. The nested LOOCV approach described above is then applied to the RAND database. The number of significant peaks selected in the “actual pathology” dataset determines the number of peaks selected in the “RAND grouping” dataset. The % of random LOOCV classified serum mass peaks and the random grouping *p* value of the classification are then calculated and plotted as exhibited in Figure 2B. The random *p* values are expected to be either non-significant or considerably larger than that obtained with the original dataset comparison when the LOOCV approach results in discrimination between groups. Loss of group discrimination upon sample randomization was also interpreted as a potential physiological basis for the original separation.

### 2.4. Test Metrics

The diagnostic value of a test/procedure is defined by its sensitivity, specificity, predictive value, and efficiency [34,35]. Test sensitivity was determined from TP/(TP+FN), where TP was the number of true positives for disease presence, and FN was the number of false negatives for disease presence. Specificity was calculated from TN/(TN+FP), where TN is the number of true negatives and FP is the number of false positives. For distinguishing TN, TP, FP, FN individuals, a test metric cut-off line (solid horizontal line in Figure 2 and other LOOCV figures) is employed. For group A (the larger % classified mass peaks group, e.g., the moderate AD group in Figure 2A), this cut-off line is determined by group A % mass peak mean minus a multiplier factor times the standard deviation (SD) for group A, and for the lower group B % mass peak mean (controls) plus the multiplier factor times the SD for group B. The multiplier factor is equal to the mean of group A minus the mean of group B divided by the SD of group A plus the SD of group B. The use of this multiplier results in two equivalent cut off lines for true pathologic separations (Figure 2A), and two independent cut off lines (using this same multiplier) when the group pathologies have been randomized (Figure 2B). This approach results in each sample, e.g., in the mild AD vs. control comparison (Figure 2A), being either correctly classified as AD (TP) or control (TN) or being wrongly classified as control (FN) or AD (FP). In the case of group randomizations, a sample can be classified as TP and FN at the same time if it lies between the two independent cut off lines, and this is what is observed in these randomizations (e.g., Figure 2B).

## 3. Results

### 3.1. Discriminating AD Patients from Control Individuals Using ESI-MS Serum Profiling

Figure 2A illustrates the application of the LOOCV/PCV serum mass peak profiling process exhibited and described in Figure 1B to help distinguish patients with mild AD (*N* = 15) from control individuals (*N* = 14). When the “% of total mild AD patient LOOCV classified serum mass peaks” is plotted versus patient number, a distribution plot is obtained in which a clear demarcation is observed between mild AD patients (dark triangles) versus control individuals (dashes). The *p* value (Student’s t test) for the moderate AD vs. control distribution difference is very low (10^−13^ range). A “cut-off” line is present in panel A which is determined from the group standard deviations (SD, see Methods), and is used to quantify false positives (FP) and false negatives (FN), none of which are present in this sample/patient discrimination; all AD samples are indicated to be true positives and all control samples are indicated to be true negatives. This cut-off line is actually composed of two lines that overlap due to similar standard deviations (SDs) plotted from the top mild AD subject group % LOOCV AD mass peak mean (−2.43%) and from the bottom control subject group % LOOCV AD mass peak mean (+2.43%). Importantly, the mild AD versus control group serum discriminatory *p* value in panel A moves toward non-significance (about 0.09) when these two subject groups (mild AD and controls) are mixed together in random fashion followed by the identical LOOCV serum mass peak analysis (panel B). Of note, upon inter-randomization of these two groups, the cut off lines now separate above and below the % means of their respective groups (control +2.43 and moderate AD −2.43, Figure 2B). Additionally, now randomization indicates that all of these samples are classified as a true positive and a false negative at the same time, which indicates that no group discrimination is occurring. This randomized LOOCV database contains the same number of mass peaks as the original pathology-specific LOOCV database, and is an attempt to remove discriminatory effects of random, undefined or unexpected influences (over-fitting). This very large increase in *p* value upon randomization observed in panel B is consistent with minimal over-fitting of the original datasets, and consistent with the presence of a physiological basis for the mild AD versus control discrimination. Panels C and D exhibit the serum mass profiling discrimination of patients with moderate AD (*N* = 16) versus control individuals (*N* = 14). The *p* value for this group distribution separation is in the 10^−14^ range, less than that observed for the mild AD vs. control comparison. As with the mild AD vs. control comparison, no false positives or false negatives are observed at these *N* values in the moderate AD vs. control comparison. Randomization of patients/subjects among the two different groups in panel C followed by the identical LOOCV serum mass peak profiling analysis resulted in little discrimination (*p* value = 0.015, panel D).

### 3.2. Discriminating Patients with Mild or Moderate AD from Control Individuals, from Each Other, and Assigning Group Designation (Control or AD) of a Blinded AD Sub-Group

Expanding the group *N* values (i.e., the serum sample numbers examined in Figure 2) and performing prospective analysis are two of the goals of future studies using this LOOCV serum profiling methodology to monitor AD. A comparison of control individuals (*N* = 14, dashes) vs. a combined mild and moderate AD patient grouping (*N* = 31, dark triangles and dark circles) in Figure 3A is a step in that direction. This comparison yields a group discriminatory *p* value in the 10^−17^ range. This lower value (vs. 10^−13 or −14^, Figure 2) is likely due to the increased *N* value of the combined AD group. The *p* value change upon inter-group sample randomization (0.16, Figure not shown) is clearly out of the significant range (<0.05), and again is suggestive of physiological differences (as exhibited by serum changes) between control individuals and AD patients included in both the mild and moderate stages. Although these are retrospective analyses exhibited in Figure 2 and Figure 3, it is possible using the present sample sizes to obtain information on whether this methodology might aid in prospective AD monitoring. Such a blinded and potential “prospective” analysis is exhibited in Figure 3B. In this panel, 10 sera samples of mild or moderate AD origin were removed (“left out”) from the 31 AD samples in panel A and the remaining 21 AD samples served as a “left in training set” for LOOCV comparison to the 14 control samples. These 10 “left out” AD samples were then analyzed against the left-in “training set” in panel B to see how their mass peak areas would partition between the AD group and the control group using the LOOCV/PCV process described above. As can be observed, all 10 AD samples (dark diamonds) partition with the training set AD samples. Such a result portends well for larger prospective studies. 

Panel C in Figure 3 exhibits the ability of the LOOCV/PCV process to distinguish the sera of mild AD patients (dark triangles) from that of moderate AD patients (dark circles); one mild AD false negative is observed. Panel D exhibits the randomization experiment between these two groups, yielding a non-significant discriminatory *p* value (0.18); no discrimination is observed between the randomized mild AD and moderate AD samples.

### 3.3. Segregation of Traumatic Brain Injury (TBI) Patient Sera with AD Patients when Compared to Control Individuals Using the LOOCV/PCV Process

The LOOCV/PCV methodology allows a group of additional “left out” samples of an unknown relationship (such as traumatic brain injury (TBI)), to be compared to an existing binary discrimination such as the AD samples vs. controls in Figure 3A. Such information can provide biochemical insight into relatedness of disease states as well as differences. This comparison is accomplished by classifying as a percentage of the individual sample mass peak areas of the “left-out” sample group against the PCVs. comprising the entire existing binary group listing of significantly differing mass peaks in the existing binary group comparison, AD vs. controls in this case. As exhibited in Figure 4A, sera samples from 13 TBI patients (United States Military Veterans [dark diamonds] who suffered a mild (loss of consciousness 1–30 min) TBI [age mean = 39.9, previously described [19], sequestered using this LOOCV/PCV procedure primarily with the AD patient grouping (open circles) versus the control grouping (dashes). It is noted that there is a significant age difference between the TBI patients and the AD/control individuals. Yet, segregation here with the AD patients versus the age-similar control individuals is consistent with age not playing a role in TBI sequestering with AD in this analysis. This result in Figure 4A indicates that the TBI state is more physiologically related to the AD state than to controls and is consistent with a previous study that elucidated important biochemical/cellular mechanistic similarities between AD and TBI, including cerebrovascular dysfunction [36].

This ESI-MS methodology was also able to discriminate a mixture of mild and moderate AD patients (dark squares) from these 13 TBI patients (dashes, panel B; non-discriminatory randomization analysis of this binary comparison in panel C). Of interest, using the “left out” procedure for these 13 TBI samples (open diamonds), they were found to segregate more with the moderate AD patients (open circles) versus the mild AD patients (11 sequestered with moderate AD vs. 2 with mild AD, panel D). To our knowledge, such apparent disease discrimination ability is not available with other biomarker platforms.

### 3.4. Distinguishing AD Patients and Control Individuals Using a Lower-Cost Desktop ESI-MS Instrument

**Figure 5:**
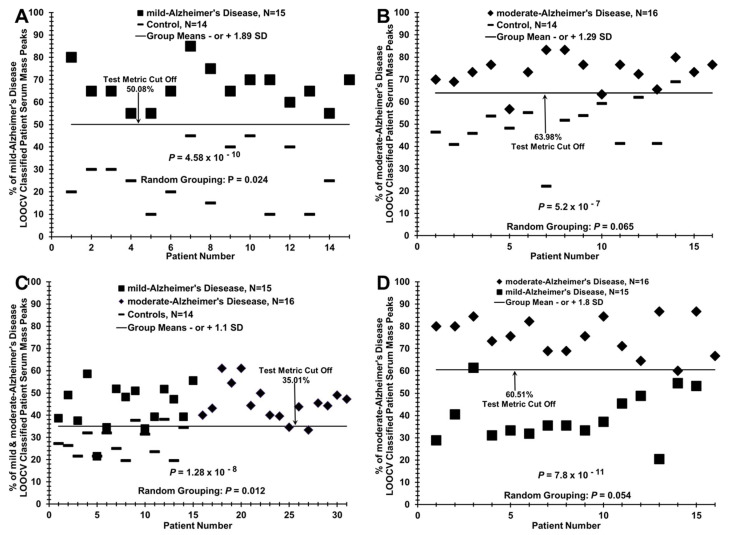
LOOCV/PCV discrimination of sera from AD patients versus control individuals, and mild AD patient sera versus moderate AD patient sera using a lower cost, desktop mass spectrometer. (**A**) Distinguishing mild AD patient sera (dark squares) from controls (dashes). (**B**) Discriminating moderate AD patient sera (dark triangles) from controls (dashes). (**C**) Distinguishing AD patient sera as a group (*N* = 31, mild [dark squares] and moderate [dark diamonds]) from controls (dashes). (**D**) Distinguishing mild AD patient sera (dark diamonds) versus moderate AD patient sera (dark squares).

The LCQ ion-trap mass spectrometer used to collect data in Figure 1, Figure 2, Figure 3 and Figure 4 is large, difficult for clinical staff to use to its maximum potential, and probably not useful in a hospital clinical lab seeing potential AD patients for the first time. Therefore, for this methodology described here to be used in such a clinical setting, a smaller, less expensive, and easy to use MS instrument would likely be needed. Additionally, potentially positive results with a lower-resolution and less accurate instrument using differing MS physics would bolster the validity of the LOOCV/PCV process. To examine the utility of such a smaller and less technology-advanced mass spectrometer to make the serum discriminations described in this study, we tested an Advion CMS Expression instrument (Advion, Inc., Ithaca, NY) in this regard. The ability of this lower-resolution, lower *m*/*z* range, and lower-cost ESI-MS instrument (with a simpler mass analyzer of differing physics) to distinguish sera from AD patient groups and from control individuals is exhibited in Figure 5. The results (panel A) show a clear demarcation between mild AD patients and controls in the % of mild AD LOOCV/PCV classified patient serum mass peaks. The *p* value for this group discrimination is in the 10^−10^ range. The same LOOCV/PCV analysis using the more accurate LCQ instrument yielded a discriminatory *p* value in the much lower 10^−13^ range (Figure 2A). Randomizing the samples between these two groups and re-analyzing their mass peaks using the LOOCV/PCV process, a *p* value of 0.024 is obtained which still is considered significant (below standard 0.05 significance value); the corresponding value for the higher-resolution LCQ instrument was 0.09 (Figure 2B). The moderate AD vs. control comparison using the less accurate instrument is not quite as good as the mild AD vs. control experiment as two false negatives and one false positive are observed (panel B); the group discriminatory *p* value is also increased from 10^−10^ to 10^−7^. LOOCV analysis of a mixture of mild and moderate AD patient sera (*N* = 31) versus sera from control individuals (*N* = 14) reveals poorer performance/group discrimination (several false positives or false negatives appearing in all groups) with the single-quadrupole mass analyzer instrument (Figure 5C, *p* value = 10^−8^) versus the more accurate LCQ ion-trap instrument (Figure 3A, *p* value = 10^−17^, same samples and *N* values). The lower-resolution instrument is able to discriminate mild AD patient sera from moderate AD patient sera, albeit with one false positive and one false negative (Figure 5D). Overall, these results show promise for the potential of easier to use and lower-cost MS technology to help distinguish and monitor AD patients in a hospital clinical lab setting.

### 3.5. Test Metric Data for AD, Control, and TBI Serum LOOCV/ESI-MS Profiling Comparisons

Table 2 summarizes the test metrics for the LOOCV data for the true-pathology group comparisons in Figure 2, Figure 3, Figure 4 and Figure 5 (figure numbers and panels listed in far right column), obtained using the ESI ion trap (panel I) or the ESI single-quadrupole (panel II) mass spectrometers. The mass analyzers in these two instruments have differing physics and electronics. Subject groups tested (Group 1 vs. Group 2) are listed in the far-left column. % LOOCV classified mass peak mean values with standard deviations (SD) for the two comparative groups are given in the separate Group 1 and Group 2 columns. Cohen’s d “effect size” values are provided in a middle panel. These values are calculated from the % LOOCV means and SDs to obtain a sense of the effect size which is a measure of the size of the observed % mean differences between the two groups under comparison [32]. Such “effect sizes” are proportional to a Cohen’s d, which is proportional to statistical power (ability to detect false-negative type II errors). The large Cohen’s d values in this study yield an estimated power of >0.90 for these LOOCV binary comparisons and bolster the reliability of the sample sizes utilized here [32,33]. The *p* values in the next column for all the LOOCV group separations using the ESI ion-trap MS instrument are very low, ranging from 10^−13^ to 10^−17^. Those *p* values for the same group comparisons then become significantly larger through the use of a lower-resolution and lower-cost single-quadrupole ESI instrument (Expression CMS, Advion, Inc.), 10^−7^ to 10^−11^. The performance of ESI-MS to classify these subjects into their true-pathology group was excellent, with a sensitivity and a specificity of 100% when the LOOCV dataset was used from the ESI-ion trap MS instrument. All these values decreased significantly using the single-quadrupole instrument with one to five false positives/false negatives appearing. These results do, however, indicate that this lower-cost instrument with reduced *m*/*z* range can still detect enough mass spectral signal differences between these two groups. This ability of two different mass spectrometers to distinguish these groups strengthens the conclusions that the biomolecules observable in the serum and differing among the study groups could possibly help in the diagnosis and monitoring of AD. Table S1 lists all the subject test metric data for the group comparisons in Table 2 with the important introduction of inter-group mixing/randomization of the study subjects in all the binary comparisons, and then re-performing the LOOCV/PCV process as utilized to generate the original true-pathology data in Table 2. This random mixing of all the subjects between the two groups in any binary comparison results in loss of any group-specific subject identifications that were observed across the data in Table 2. The “effect” sizes become much smaller and the “discriminatory” *p* values become much larger. These randomization/mixing results compared to the true-pathology results are consistent with minimal over-fitting of the large datasets and support the presence of a physiological basis for the serum LOOCV differences observed (Figure 2, Figure 3, Figure 4 and Figure 5) between the control and AD study groups, even using the lower-cost MS instrument.

### 3.6. Phenotype Assessments of AD Patients Using Tandem MS/MS of Serum Peptide/Proteins, and Bioinformatic Cell Pathway/Disease Mechanism Analysis

Tandem MS/MS analysis of 8 mild AD patient sera and 8 sera from control individuals, in the range of 900–1008 *m*/*z*, was employed to examine potential phenotypic peptide/protein differences and similarities between these patient/control groups. This 900–1008 range was empirically shown previously to provide ample ionization for serum MS/MS peptide/polypeptide identifications [19,20,21]. The mild AD vs. control comparison was chosen to examine potential differences between non-disease controls and AD disease early onset. To focus on peptides/proteins with larger differences between the two groups, a set of 154 different peptides/proteins showing at least a 2-fold difference in number of positive subject sera (3 or more out of the total 8 sera per group) and a 1.5-fold difference or greater in peptide sera “hits” between the mild AD and control groups were chosen and exhibited in Table 3 (differences in mild AD greater than controls) and Table 4 (differences in controls greater than mild AD). A MS/MS “hit” (single-peptide identification) ratio between the two groups of at least a rounded off value of 1.5 was employed. A total of 247 different peptides/proteins meet the 3 or more sera criteria before this sera/hit number filtering (unfiltered) and are exhibited as supplemental Tables S2 and S3. The MS/MS methodology employed here is mainly identifying serum peptides and polypeptides (peptidome) and not intact larger proteins. This serum peptidome has been proposed to be more disease specific than organismal proteomes [37]. It is known that peptides can pass freely as well as pass in regulated fashion through the blood–brain barrier [38] Thus, this methodology could be gleaning biochemical phenotype information directly from the brain affected by AD. Two of the more prevalent peptides/proteins evident are SSPO (SCO-spondin, sub commissural organ spondin, present in 8 out of 8 mild AD patient sera, Table 3) and VWF (von Willebrand factor, present in 6 out of 8 mild AD patient sera, Table 3). SSPO is involved in neurogenesis and neuronal survival [39,40], and VWF is involved in endothelial cell vascular function and inflammation [41,42]. To begin to ascertain potential biochemical function and phenotype of all the proteins in Table 3 and Table 4, a manual PubMed/Medline search (using the “protein symbol and disease” search labels) of the 154 differentially expressed peptides/proteins was performed. Some of the different functional phenotypes observed for these proteins and their percentages in Tables (out of 154 peptides/proteins) are listed by numbers 1 through 7 in Table legends: 34%—Alzheimer’s disease/dementia/amyloidosis (shaded cells in Tables); 32%—neurogenesis; 32%—inflammation/neuroinflammation; 18%—vasculature; 16%—ion channels; 14%—neuronal cell death; 9%—brain injury/CNS trauma/blood–brain barrier.

**Figure 6:**
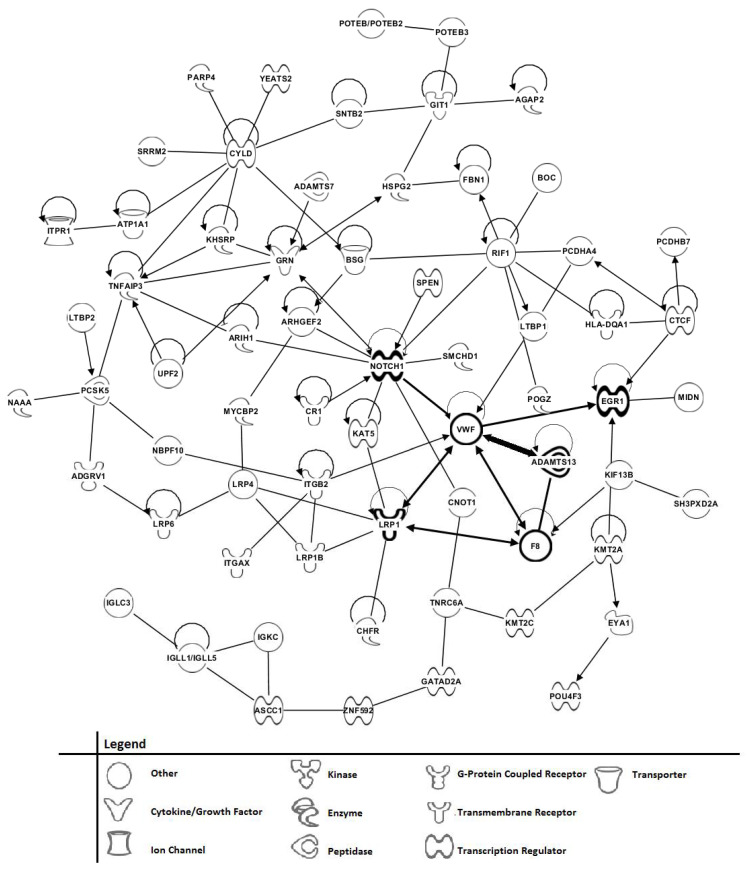
Potential protein IPA connections (no functional considerations) in mild Alzheimer’s disease as determined by serum tandem MS/MS peptide/protein analysis. Arrows indicate a direct scientific literature connection and lines indicate indirect literature connections. The bolded arrow indicates the VWF/ADAMTS13 protein (proteins in bold) axis, and the darkened arrows indicate other potential protein connections to this axis (protein names in bold). The protein function symbol legend is at the bottom of the figure.

Figure 6 depicts proposed connections (without invoking function, arrows imply direct connection) involving a grouping of proteins that possibly are involved in mild (earlier onset) AD. These data were gleaned from scientific literature information using Ingenuity Pathway Analysis software (IPA, Qiagen, Inc. [28]) of the 154 proteins meeting the 2-fold or more sera difference in Table 3 and Table 4. This observed grouping encompasses 66 out of the 154 proteins or 43 %, and imply functional relatedness to AD early-onset (mild AD compared to controls). Observed in Figure 6 are 13 major protein nodes with 5 or more connections (including autoregulation). These include (in order of 11 to 5 connections): NOTCH1 (Notch receptor 1, NOTCH signaling, vasculature permeability [43]; CYLD (cylindromatosis, lysine 63 deubiquitinase, dementia [44]; RIF1 (Rap1-Interacting Factor 1, telomere and chromosome integrity [45]; VWF (von Willebrand Factor, vascular inflammation, dementia [42,46]; GRN (Granulin precursor, secreted growth factor progranulin, Parkinson’s disease, dementia [47,48]; low-density lipoprotein receptor [LDLR]-related protein 1 (LRP1, dementia, blood–brain barrier, AD [49]; TNFAIP3 (Tumor Necrosis Factor Alpha Induced Protein 3, A20 protein, autoimmunity, NFkB regulatory protein [50]; CTCF (CCCTC-binding Factor, chromatin structure, epigenetics, intellectual disability [51]; EGR1 (Early Growth Response gene 1, neurodegeneration, neuroinflammation [52]; F8 (factor VIII, bleeding disorders, VWF connection [53]; GIT1 (G Protein-Coupled Receptor Kinase-Interacting Protein-1), regulator of neuronal function, brain development, memory [54]; ITGB2 (Integrin subunit β 2, immunomodulation, CD18 protein, brain immune homeostasis, aging brain [55]; PCSK5 (Proprotein Convertase Subtilisin/Kexin type 5, lipoprotein metabolism, cognitive impairment [56].

An interesting tetrad of connections exists here composed of VWF, LRP1, F8, and ADAMTS13 (A Disintegrin-like And Metalloprotease with Thrombospondin type 1 motif, 13). ADAMTS13 is a metalloprotease that cleaves VWF [57]. This VWF/ADAMTS13 axis connection in the lower right of Figure 6 (bolded), again proposed with no consideration of function, is of interest since changes in this axis appear to regulate endothelial cell vascular function and inflammation [42,57]. The clotting factor F8 connection (darkened) is part of this axis, physically interacting with both VWF and ADAMTS13 [46]. Additionally, included in this axis is the LRP1 connection (darkened), which was previously shown to help clear amyloid β from the endothelial cell cerebral vasculature through the blood–brain barrier (BBB) [58]. A connection here (darkened) to this WVF/ADAMTS13 axis is reported for NOTCH1 which has roles in vascular epithelium, vascular disorders, and BBB function [43]. A connection (darkened) of this axis to EGR1 is also noted. This protein is a member of the immediate early gene (IEG) transcription factors and plays a role in memory formation, possibly through synaptic transmission and vesicular transport; EGR1 can also play a role in neurodegeneration [59].

**Figure 7:**
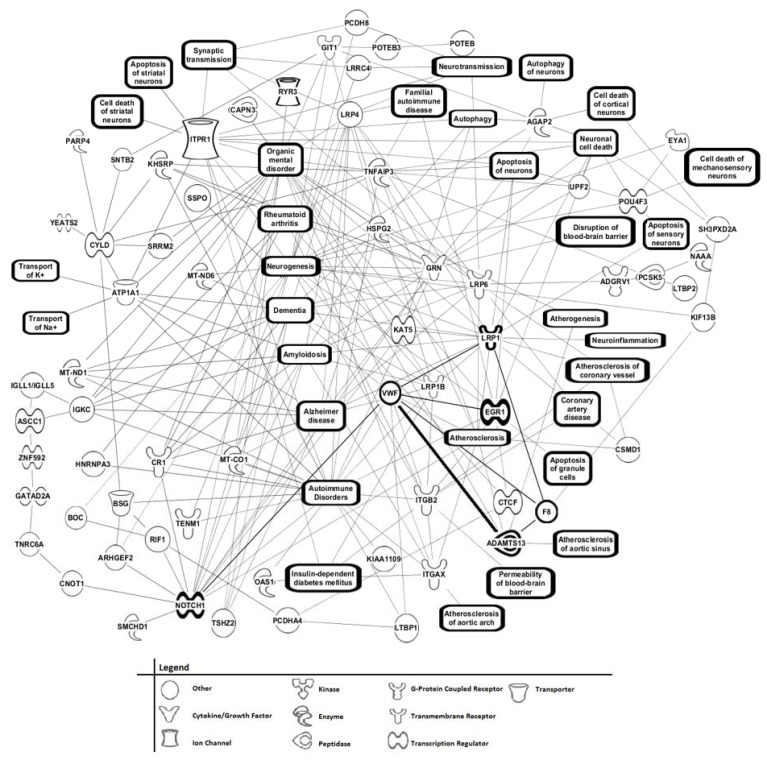
IPA of *m*/*z* “range” MS/MS serum data from mild AD patients vs. control subject comparison using top 119 peptides/proteins from Table 3 and Table 4. IPA *m*/*z* range MS/MS serum data analysis for mild AD patients vs. controls using top 119 peptide/proteins from Table 3 and Table 4. Ingenuity Pathway Analysis (IPA), Qiagen, Inc.) of the 119 peptides/proteins exhibited in Table 3 and Table 4 having a 2x difference in positive sera number between mild AD patients and controls, and a 1.5x difference in MS/MS “hit” [single-peptide identification] ratio between the two groups. The protein function symbol legend is at the bottom of the figure.

The peptides/proteins expressed differently in sera between mild AD patients and control individuals as exhibited in Table 3 and Table 4 were also analyzed for function and phenotype using IPA to potentially identify affected cellular/biochemical pathways and related phenotype/pathological states (Figure 7, top 119 [top 60 from Table 3 plus LTB1, HSPG2, RYR3, MT-ND6---AD related; top 50 from Table 4 plus PTPRQ, TSHZ2, LRP6—AD related; KIF13B, RGS12—ion channel related] out of the 154 proteins in Table 3 and Table 4 present).

IPA is used in this context to predict cellular pathways that are possibly changing based on altered gene expression parameters [28]. In this case, serum peptidome changes are utilized which are valid gene expression markers for disease states [37]. The serum peptidome is hypothesized to better reflect an organism’s systemic phenotype and associated disease states than individual organ proteomes or the proteome in general. Observed in the left side of Figure 7, major networks of cellular/biochemical pathways/phenotypes (and their associated proteins) possibly affected in the mild AD vs. control IPA comparison include the AD, dementia, amyloidosis, and autoimmunity related proteins mitochondrial (MT) NADH dehydrogenase subunit 6 [MT-ND6], MT-ND1 (NADH dehydrogenase subunit 1), and MT-CO1 (Cytochrome c oxidase subunit 1). These protein levels were previously observed to be altered in the peripheral blood of early AD patients [60]. CR1 (Complement receptor 1) and its network appears to be involved in these same phenotypes and was previously implicated in AD and amyloidosis [61]. A NOTCH1 signaling network is present here and connected to these same phenotypes. In the middle section of Figure 7, the low-density lipoprotein receptor-related proteins LRP1, LRP1B, LRP4, and LRP6 are evident with their connections to AD-related phenotypes being apparent as well as to vasculature pathologies (e.g., atherosclerosis). LRP6 and LRP4 were previously associated with AD and coronary artery disease [62]. LRP1B was reported associated with AD and schizophrenia [63].

In the middle of Figure 7, the physiological networks/phenotypes involving the GRN protein include AD, amyloidosis, dementia, apoptosis, neuronal cell death, and blood–brain barrier disruption. ITGB2 has roles in autoimmunity, diabetes, and atherosclerosis. As in Figure 6, the connections composed of NOTCH1, VWF, and LRP1, with off-shoots to ADAMTS13, EGR1, RIF1, GRN, and F8 are observed in this middle IPA section of Figure 7. The VWF/ADAMTS13 axis connection (bolded) is connected to dementia, mental disorders, autoimmunity, and atherosclerosis, are particularly interesting since changes in this axis appear to regulate vascularization and inflammation. This axis is also connected to NOTCH1 (darkened), F8 (darkened), LRP1 (darkened), and EGR1 (darkened). Table 3 and Table 4 indicate that in mild AD patients, VWF is elevated and ADAMTS13 is depressed (6 out of 8 AD samples had detectable VWF and 0 out of 8 had detectable ADAMTS13; in controls 1 out of 8 samples had detectable VWF and 3 out of 8 samples had detectable ADAMTS13). Such an imbalance in the VWF/ADAMTS13 axis is indicative of a vascular inflammatory state in AD. Moreover, the reduced plasma ADAMTS13 activity and increased plasma VWF are risk factors for the development of other arterial and inflammatory diseases, including myocardial infarction and stroke [42,57]. In the upper right side of Figure 7 a network of proteins involved in autophagy, apoptosis, and neuronal/sensory cell death phenotypes is observed (AGAP2, UPF2, POU4F3). AGAP2 (ArfGAP with GTPase domain, ankyrin repeat and PH domain 2; phosphatidylinositol 3-kinase enhancer—PIKE) was previously assed to have roles in neuron apoptosis and cell death [64]; here it is implicated in autophagy as well. UPF2 (Up-Frameshift Suppressor 2) is involved in mRNA degradation [65], and POU4F3 (POU Domain Class 4 Transcription Factor 3) is a transcription factor involved in survival of sensory and motor neurons, apoptosis, and autophagy [66,67]. In the lower right of this figure is a CSMD1 (CUB and Sushi Multiple Domains 1) hub with connections to atherosclerosis phenotypes. The CSMD1 gene was previously related to neurogenesis, cognition, immunity, inflammation, neuropsychology, and monoamine metabolism of SCZ [68].

## 4. Discussion

Biomarker approaches are excellent tools for monitoring and understanding pathologies such as Alzheimer’s disease (AD), as well as aiding in treatments through identification of potential therapeutic targets. Although biomarker progress on AD has been substantial, recent advances in large input/throughput approaches (e.g., genomic, transcriptomic, proteomic, metabolomic) show additional promise in monitoring and understanding AD. It is important in these “omic” approaches to examine potential roles of cellular/pathophysiological networks alluded to from data collection as diseases such as AD are complex. Developing such biomarker and cell network approaches, using readily available bodily sources such as peripheral blood, would be helpful. The purpose of the present study was to examine a novel methodology to see whether it could help identify and monitor patients with mild or moderate AD. The electrospray ionization mass spectrometry (ESI-MS) approach is straight-forward and simplified by using unfractionated serum to help distinguish and monitor AD patients from each other and from control individuals. The hypothesis of this methodology is that AD induces organs and tissues (including the brain) to release/shed specific biomolecules into the peripheral blood, e.g., peptides, involved in the disease state (primarily or secondarily) as well as biomolecules involved in specific systemic responses to that disease state. Examination of biomolecules in peripheral blood, e.g., peptides/proteins that change with AD, has the potential to provide diagnostic, phenotypic, mechanistic, and therapeutic insights into this disorder. The serum peptidome is a valid gene expression entity for study and was shown to correlate with specific disease states [19,20,21,37].

ESI-MS combined with the novel LOOCV/PCV approach in this study identified serum mass peak areas changing significantly upon comparison of patients with mild or moderate AD and upon comparison to control individuals. All study subjects were reasonably matched with respect to age and sex. Randomization of serum samples between these groups undergoing comparison followed by this LOOCV/PCV mass peak analysis resulted in loss of group-specific discrimination ability, suggesting a physiological basis for the AD and control binary group discriminations. The ability to distinguish these groups is likely due to the large number of different identifiers (94 to 110 mass peaks used in the LOOCV Figure 2A mild AD group vs. control discrimination) as the larger the number of such identifiers the greater the disease discriminatory of a platform [19,22]. Of added importance, this LOOCV/PCV methodology was also able to distinguish sera from patients with mild from moderate AD patients (Figure 3C). These group discriminatory results were substantiated using an ESI-MS instrument with a mass analyzer of differing physics (single quadrupole), lower resolution, and easier use (Figure 5). This result indicates the potential of this methodology being applied in hospital clinical lab diagnostic setting. These overall results support the hypothesis that early-stage (mild) and moderate stage AD induces biomolecular alterations that are reflected in the peripheral blood and can have a role in identifying these specific clinical groups. More specifically, these results indicate that this ESI-MS approach described has potential for monitoring and understanding early AD as well as aiding in therapeutic development, thus warranting further study. Of note, all of the LOOCV analyses presented in this study exhibit quite large Cohen’s d “effect sizes” (proportional to mean differences and standard deviations of the group-specific % LOOCV classified mass peaks) in the exhibited binary comparisons. Such “effect sizes” are proportional to statistical power (ability to detect type II errors, false negatives). The large Cohen’s d values in this study (Table 2) yield an estimated power of >0.90 for these LOOCV binary comparisons and bolster the reliability of the relatively small sample sizes utilized here [32,33]. These results portend well for future studies validating these observations with larger sample groups, as well as prospective-type completely blinded sample studies. It is further noted in this regard that the methodology was able to identify a “blinded” set of ten “left out” AD serum samples as AD samples (Figure 3B). An important addition to this study is the inclusion, for comparative purposes to AD patients, sera from a group of traumatic brain injury (TBI) patients that were described in a previous study on TBI [19]. Similarities between AD and TBI pathologies were previously noted, especially from a common cerebrovascular dysfunction viewpoint [36]. Using the LOOCV/PCV sera mass peak analytical procedure, this group of TBI patients (*N* = 13) segregated with the AD patients in the AD patient comparison to control individuals, Figure 4A. This suggests that with respect to apparent physiological changes responsible for these LOOCV serum sample separations, the TBI condition is more related to the AD condition than to non-dementia control individuals. Importantly, a separate LOOCV analysis was able to discriminate directly the TBI patients from the AD patients (Figure 4B) indicating and suggesting that different disease conditions are in fact present.

Besides these disease group discriminations, the MS methodology employed may be able to assist in understanding biochemical mechanisms in early (mild) AD development, by identifying potential cell/biochemical pathway involvements, of novel biomarkers, and therapeutic targets. Of the serum mass peaks observed in this study, for example in Figure 1B, many are between approximately 500 and 1200 *m*/*z*, and likely include host tissue/organ exoprotease activities and other cell/tissue signaling activities resulting from the lower mass peptide “serome”, biomolecules [21,22]. To assist in identifying physiologically related differences in this complex biomolecular milieu, MS/MS structure determinations were performed. At the ionization energies employed here, the intact larger proteins are not likely to be fragmented, the MS signal may indicate only existing peptides and polypeptides. The identification of changing observations of peptides and biochemical pathways could be helpful in understanding underlying AD disease mechanisms, especially at the earlier stages, and developing novel diagnostic biomarkers and therapeutics. For these purposes of protein/peptide identification, a range analysis (900–1008 *m*/*z*) was conducted, revealing a prominent mild AD phenotype with 52 of the 154 different proteins (34%) with known associations to AD/dementia/amyloidosis, Table 3 and Table 4. Other prominent phenotypes gleaned from this list in descending order below this dementia class are neurogenesis, inflammation/neuroinflammation, vasculature, ion channels, neuronal cell death, and brain injury/blood–brain barrier.

When the top 119 out of these 154 proteins were subjected to IPA bioinformatics software, a number of prominent phenotypic “hubs” of protein pathway connections appeared which can provide further molecular insight into AD (Figure 7). These cell/biochemical pathways are hypothetically changing in the brain and/or changing systemically based on the serum gene expression changes reflected in differing amounts of the peptides between the mild AD and control groups provided in Table 3 and Table 4. Connecting to Alzheimer’s disease, dementia, and amyloidosis hubs include prominent AD-associated proteins such as the low-density lipoprotein receptor-related proteins LRP1, LRP1B, LRP4, and LRP6. Lipids, lipoproteins, and their receptors play important roles in normal brain health as well as in brain pathologies such as AD [69]. A group of mitochondria proteins related to energy generation were observed in these same pathways/hubs that were previously shown to have AD/dementia/amyloidosis associations including MT-ND1, MT-ND6, and MT-CO1. Impaired mitochondrial energy metabolism appears to be a hallmark of AD, often preceding early onset of the disease [70]. Other important hubs and pathway connections include the GRN protein, which bridges AD pathways to neuronal cell death and blood–brain barrier (BBB) disruption, other hallmarks of this dementia [49]. The AGAP2 protein forms pathway connections to autophagy, apoptosis, and neuronal cell death, again possible phenotypic features of AD. Notably there exists a VWF/ADAMTS13 axis hub in Figure 7 connecting dementia and atherosclerosis phenotypes. VWF has roles in blood vessel development and vascular injury, repair, and inflammation. ADAMTS13 is a zinc-containing metalloprotease that cleaves von Willebrand factor (VWF). Changing levels of these two proteins relative to each other is proposed to have roles in hemostasis, aberrant endothelial cell/blood vessel vascularization, and inflammation when VWF levels significantly exceed ADAMTS13 levels. Reduced plasma ADAMTS13 and increased plasma VWF are also risk factors for the development of arterial and inflammatory diseases, including myocardial infarction and ischemic stroke. In the present study, elevated levels of VWF were observed in the sera of mild AD patients compared to controls, and depressed levels of ADAMTS13 were observed in mild AD patients compared to controls, consistent with this imbalance possibly having a role in AD development. Such an imbalance of these two proteins was previously observed in the plasma of dementia patients, including a group with AD, and was proposed to be associated with increased risk for dementia [46]. These observations about this axis in the present study, alluding to its importance in a group of AD patients, could provide additional mechanistic clues for the previous study suggesting that the axis and associated phenotypic changes have roles in dementia development. Of added interest, the blood-clotting protein F8 (factor VIII) is also connected to this axis and hence to these phenotypes in Figure 6 and Figure 7. Factor VIII is known to have physical interactions with both VWF and ADAMTS13, and promotes the latter’s cleavage of VWF [71]. The present study proposes this axis be extended to include F8, and that the VWF/ADAMTS13/F8 axis has a role in AD development. In addition, LRP1 is proposed in the present study to also be included in this cerebral vascular axis (Figure 6 and Figure 7). Aberrant levels of LRP1 in brain vascular endothelial cells were previously linked to neurodegeneration, human brain aging, and AD [72].

VWF was the second most observed peptide/protein in sera of our mild AD patients, and SSPO was the most prevalent peptide/protein found (8 out of 8 mild AD patients, Table 3). Although VWF has numerous AD annotations in the scientific literature, no connections to AD were found for the SSPO protein in PubMed (personal communication). SSPO is a large multidomain protein of the extracellular matrix (ECM), and is involved in the development of brain commissural fibers and is neuroprotective against oxidative stress-induced cell death [38]. SSPO was previously identified as having a possible role in Parkinson’s disease (PD) pathogenesis [39]. The exact role of SSPO in PD pathogenicity is undetermined; literature citations indicate that SSPO is involved in neuronal survival, aggregation, and neurite extension [38]. An sspo gene mutation was previously associated with depressive disorder (DD) [73]. Investigating SSPO as a potential novel AD biomarker appears to warrant further study. 

With respect to potential weaknesses of this study, the relatively small sample sizes are potentially problematic. However, the overall LOOCV process produces large ‘effect” sizes (differences between the means and standard deviations between any binary group comparison), which yield high statistical power from reduced sample sizes. Another potential problem present in all Human studies is accuracy of diagnosis, in this AD. Our behavioral neurologist (L.A.H.) is confident in these AD clinical diagnoses, since published procedures were used to make the diagnosis [23,24], and other types of dementia were screened for and ruled out clinically. Bolstering our confidence in our results using our given sample sizes and clinical diagnoses, another group came to the same conclusion about the presence of an imbalanced VWF/ADAMTS13 axis using a larger group of AD as well as non-AD dementia patients [46]. Importantly, we came to a similar conclusion about this axis using completely different methodology.

Overall, results from this study, especially the observed VWF/ADAMTS13 axis and its imbalance, support the theory that impaired vascularization and blood flow are important determinants in AD and its development in the brain [74,75]. Elevated VWF levels would promote thrombotic events that could impinge on blood vessels [76]. Such impairment would reduce blood flow and proper clearance of the amyloid β and Tau proteins which build up in the AD brain causing neurodegeneration [77]. Low blood pressure, which would correlate with reduced vascular flow, is also a risk factor for AD development and progression, especially for adults age 65 and older [78]. Individuals with cardiovascular disease components, including impaired vascular flow and diabetes, have increased risk for developing AD [79]. Additionally, consistent with this theory is the observation that regular exercise appears to possibly prevent AD as well as benefit AD patients, possibly due to improved vascular blood flow [80].

## 5. Conclusions

A novel serum MS profiling platform procedure (LOOCV/PCV) was developed that was able to distinguish AD patient groups (mild vs. moderate) and a control group. Additionally, the procedure was able to correctly identify a blinded AD group, and show similarities and differences between a mild TBI patient group and AD. Although sample sizes utilized are relatively small, large Cohen’s *d* effect sizes were observed with resultant high power bolstering the sample size validity. Serum peptide/protein identifications suggested that an imbalanced VWF/ADAMTS13 axis may have a role in AD development. Such an axis may have biomarker potential in AD. Other proteins potentially involved in this axis in AD include LRP1, NOTCH1, F8, and EGR1, which could also have biomarker potential. Protein SSPO also appears to be a potential serum biomarker for mild AD. Bioinformatics analyses indicate that potential pathways involved in AD development include neuronal cell death, vasculature, neurogenesis, AD/dementia/amyloidosis, neuroinflammation, autoimmunity, autophagy, atherosclerosis, and blood–brain barrier dysfunction.

## Figures and Tables

**Table 1:** Demographic and clinical information about study volunteers.

Panel I, Demographic Information about Study Participants				
	Control (*n* = 14)	Mild AD * (*n* = 15)	Moderate AD * (*n* = 16)	All AD * (*n* = 31)
Age, mean (yrs range)	74.3 (59−84)	74.9 (56−90)	77.3 (61−88)	76.2 (56−90)
Sex, men/women	7/7	5/10	7/9	12/19
Race **, AA (C)	0 (14)	0 (15)	1 (15)	1 (44)
Smoking ***, men (women)	0 (2)	3 (1)	0 (3)	3 (4)
Clinical Dementia Rating (CDR):	0	0.5 < CDR ≤ 1	1< CDR ≤ 2	>0.5
Mini-Mental State Exam (MMSE) (mean)	29.14	22.6	15.5	18.9
Instrumental Activities of Daily Living (IADL)	Normal	Below Normal	Below Normal	Below Normal
**Panel II, Inclusion/Exclusion Criteria for Participants**				
**Inclusion Criteria (General)**	**Exclusion Criteria (General)**		**Control Inclusion**	**Alzheimer’s Inclusion**
- Age 50–90 years - Ability to read and speak English - CDR in range of 0–2.0 - Community-dwelling adult - Patients and caregivers were recruited from Dr. Hershey’s practice at OU Physicians	- Receptive or expressive aphasia - Bed ridden or chair bound - Legally blind - Clinical Dementia Rating (CDR) score of 3.0 - MMSE < 10/30 - Severe depression, anxiety - (NPI > 5/12) - Dementia other than AD - Mild cognitive impairment (MCI) (0 < CDR ≤ 0.5) - OU staff or employees - Neuropsychiatric Inventory (NPI): used to screen out excessive psychopathology (NPI > 5/12 excludes patient from study)		- MMSE > 24/30 - Normal IADLs	- Memory loss - Exclusion of delirium - Functional Impairment in IADLs - AD criteria, according to McKhann et al 2011 [24]

* AD, Alzheimer’s disease. ** AA, African American; C, Caucasian. *** 1 or more cigarettes/day.

**Table 2:** LOOCV Test Metrics for “true-pathology” group comparisons.

ESI-Ion Trap MS Group 1 vs. Group 2	Mean (SD) Group 1	Mean (SD) Group 2	*“Effect* *Size”*	*p* Value	N Group 1:Group 2	Sensitivity	Specificity	Figure#
moderate-Alzheimer’s disease vs. Control, *N* = 14	62.73% (7.34%)	28.07% (6.85%)	4.88	6.06 × 10^−14^	16:14	1	1	Figure 2A
mild-Alzheimer’s disease vs. Control, *N* = 14	63.67% (5.01%)	37.29% (5.86%)	4.83	4.24 × 10^−13^	15:14	1	1	Figure 2C
Alzheimer’s disease, *N* = 31 vs. Control, *N* = 14	49.81% (3.81%)	28.53% (3.50%)	5.81	3.63 × 10^−14^	31:14	1	1	Figure 3A and Figure 4A
mild-Alzheimer’s disease vs. moderate-Alzheimer’s disease	67.33% (4.92%)	42.66% (5.41%)	0.77	3.25 × 10^−14^	15:16	0.93	1	Figure 3C and Figure 4D
Training set: Alzheimer’s disease vs. Control	55.25% (3.15%)	20.09% (2.36%)	12.63	1.24 × 10^−24^	21:14	1	1	Figure 3B
mild and moderate-Alzheimer’s disease vs. TBI	64.12% (4.17%)	26.78% (6.13%)	7.12	1.53 × 10^−14^	18:13	1	1	Figure 4B
**ESI-Single-Quad MS** **Group 1 vs. Group 2**	**Mean (SD)** **Group 1**	**Mean (SD)** **Group 2**	***“effect*** ***size”***	***p*** **Value**	**N Group 1:Group 2**	**Sensitivity**	**Specificity**	**Figure#**
mild-Alzheimer’s diseasevs. Control, *N* = 14	66.67% (8.80%)	26.07% (12.74%)	3.70	4.58 × 10^−10^	15:14	1	1	Figure 5A
moderate-Alzheimer’s diseasevs. Control	73.14% (7.11%)	49.35% (11.36%)	2.51	5.12 × 10^−7^	16:14	0.93	0.86	Figure 5B
Alzheimer’s diseasevs. Control, *N* = 14	44.82% (8.92%)	27.91% (6.45%)	2.17	1.28 × 10^−8^	31:14	0.93	0.71	Figure 5C
moderate-Alzheimer’s disease vs. mild-Alzheimer’s disease	75.56% (8.35%)	39.42% (11.14%)	3.67	7.75 × 10^−11^	15:16	0.93	0.94	Figure 5D

Leave one out cross validation (LOOCV); mass spectrometer (MS); standard deviation (SD); effect size measured by Cohen’s d; p value measured by Student’s t-test; true negative (TN); false negative (FN).

**Table 3:** Top 90 peptides/proteins from the mild AD > greater than control subjects with 2x or better sera differences and 1.5x or better MS/MS “hit” numbers.

Mild AD > Control, 2x Sera, 1.5x Hits								
	Symbol	Mild: Control		Symbol	Mild: Control		Symbol	Mild: Control
		[#Sera(#Hits)]			[#Sera(#Hits)]			[#Sera(#Hits)]
1	SSPO ^2,3,7^	8(162): 4(57)	31	STXBP2 ^2,3^	3(40): 0(0)	61	ZC3H4	3(12): 0(0)
2	VWF ^1,2,3,4,5,6^	6(158): 1(15)	32	PKD1P6	3(39): 0(0)	62	NOXP20	3(11): 0(0)
3	IGKC ^1,2^	5(130): 1(3)	33	KMT2E ^2,3^	3(37): 0(0)	63	ARIH1	3(9): 0(0)
4	F8 ^1,2,3^	4(84): 0(0)	34	SRRM2 ^1^	3(37): 0(0)	64	OIT3 ^4^	3(8): 0(0)
5	CR1 ^1,2,3,5,7^	4(30): 0(0)	35	CNOT1	3(35): 0(0)	65	IGLC3	3(259): 1(2)
6	UPF2 ^2,3^	4(30): 0(0)	36	POU4F3 ^2,3^	3(35): 0(0)	66	CHFR ^2,3,6^	3(169): 1(22)
7	LTBP2	4(66): 1(5)	37	CYLD ^2,3,4,6,7^	3(33): 0(0)	67	POGZ ^3^	3(126): 1(26)
8	ADGRV1 ^3^	4(66): 1(15)	38	CAPN3 ^4^	3(32): 0(0)	68	AGAP6 ^3^	3(119): 1(3)
9	SVEP1	4(56): 1(7)	39	ITGAX ^1,2,6^	3(32): 0(0)	69	LTBP1 ^1^	3(118): 1(25)
10	LRP4 ^2,3^	4(42): 1(5)	40	SMCHD1	3(30): 0(0)	70	HSPG2 ^1,3,5,6^	3(73): 1(3)
11	ZNF469	4(37): 1(5)	41	GIT1 ^1,2,4,5,6,7^	3(29): 0(0)	71	PRUNE2	3(59): 1(7)
12	FBN1 ^1,2,3,4,5^	4(28): 1(14)	42	ACACB ^1,2,3^	3(27): 0(0)	72	FRMPD1	3(32): 1(9)
13	MT1B	4(17): 1(3)	43	C5orf42 ^4^	3(26): 0(0)	73	RYR3 ^1,2,3,4,6^	3(32): 1(15)
14	MT-ND1 ^1^	4(127): 2(25)	44	KHSRP ^2^	3(26): 0(0)	74	MIDN ^2,3,5,7^	3(27): 1(6)
15	OTOGL	4(74): 2(25)	45	OR7G1	3(26): 0(0)	75	CELSR2 ^3^	3(25): 1(4)
16	NOTCH1 ^1,2,3,4,5,6,7^	4(39): 2(22)	46	ASCC1 ^2^	3(25): 0(0)	76	SEC24C ^1,7^	3(25): 1(5)
17	LRP1 ^1,2,3,4,5,6,7^	4(34): 2(13)	47	MFRP	3(25): 0(0)	77	NBPF10 ^1,7^	3(24): 1(3)
18	DLGAP5 ^7^	3(115): 0(0)	48	SSC5D^2^	3(25): 0(0)	78	FREM2	3(24): 1(7)
19	BSG ^1,2,7^	3(72): 0(0)	49	HNRNPA3 ^1,3^	3(23): 0(0)	79	KMT2C ^3^	3(24): 1(11)
20	NFXL1	3(59): 0(0)	50	RXFP3 ^1,6^	3(23): 0(0)	80	MT-ND6 ^1^	3(22): 1(8)
21	KIAA119	3(55): 0(0)	51	AGAP2 ^1,7^	3(22): 0(0)	81	PDZD2	3(22): 1(9)
22	TNRC6A ^6^	3(53): 0(0)	52	NAAA ^1,2^	3(22): 0(0)	82	ADAMTS6	3(19): 1(3)
23	ATP1A1 ^1,2,4,6^	3(52): 0(0)	53	MEGF11	3(21): 0(0)	83	MALRD1 ^3^	3(17): 1(8)
24	SPINK5 ^2^	3(51): 0(0)	54	ATMIN ^3,6^	3(19): 0(0)	84	PCDHB7	3(16): 1(7)
25	PCDH8 ^4^	3(46): 0(0)	55	EGR1 ^1,2,3,4,5,6,7^	3(18): 0(0)	85	SPEN ^3,7^	3(15): 1(5)
26	ARHGEF2 ^2,3^	3(45): 0(0)	56	GOLGA2P11	3(16): 0(0)	86	MYCBP2 ^3^	3(15): 1(6)
27	CFAP221	3(45): 0(0)	57	LOC4682	3(16): 0(0)	87	HLA-DQA1	3(14): 1(5)
28	EYA1 ^3,6^	3(45): 0(0)	58	TENM1	3(15): 0(0)	88	TRIP11	3(13): 1(5)
29	SARDH	3(44): 0(0)	59	PCDHA4 ^1^	3(13): 0(0)	89	POLA1	3(9): 1(3)
30	RALGAPA2 ^2^	3(41): 0(0)	60	TNFAIP3 ^2,3^	3(13): 0(0)	90	CDCA2 ^3^	3(8): 1(3)

^1^—Alzheimer’s disease/dementia/amyloidosis (shaded); ^2^—inflammation/neuroinflammation; ^3^—neurogenesis; ^4^—ion channels; 5—brain injury/trauma/blood–brain barrier; ^6^—vasculature; ^7^—neuronal cell death.

**Table 4:** Top 64 peptides/proteins from the control > mild AD subjects with 2x or better sera differences and 1.5x or better MS/MS “hit” numbers. Control > mild, 2x sera, 1.5x hits.

	Control > Mild AD, 2x Sera, 1.5x Hits							
	Symbol	Control: Mild		Symbol	Control: Mild		Symbol	Control: Mild
		[#Sera(#Hits)]			[#Sera(#Hits)]			[#Sera(#Hits)]
1	RIF1 ^6^	5(105): 2(43)	23	ADAMTS13 ^1,2,6^	3(29): 0(0)	45	BOC ^3^	3(14): 0(0)
2	ZNF142	4(67): 0(0)	24	AMBP ^7^	3(28): 0(0)	46	LRRC4	3(14): 0(0)
3	BAZ1A	4(39): 0(0)	25	PDE9A ^1,5^	3(27): 0(0)	47	SNTB2	3(11): 0(0)
4	LAMA3 ^1,2^	4(46): 1(2)	26	SH3PXD2A ^1,5^	3(27): 0(0)	48	MC1R ^1,2,4,5,6^	3(10): 0(0)
5	NAV2 ^1,3,4^	4(70): 2(34)	27	STK36 ^3,4^	3(27): 0(0)	49	RAB3GAP2 ^1^	3(9): 0(0)
6	OBSCN	4(32): 2(17)	28	STXBP5L ^1^	3(26): 0(0)	50	POTEB ^2^	3(3): 0(0)
7	MT-CO1 ^1,6^	4(22): 2(9)	29	PLXNB2 ^2,3,6^	3(25): 0(0)	51	CYP4F11	3(143): 1(8)
8	LRP1B ^1,6^	4(17): 2(6)	30	ZNF592 ^1^	3(23): 0(0)	52	KMT2A ^3^	3(124): 1(9)
9	PITRM1 ^1,4,7^	3(94): 0(0)	31	CLDN7 ^2,6^	3(22): 0(0)	53	ADAMTS7 ^2,6^	3(50): 1(18)
10	PCSK5 ^1,2^	3(56): 0(0)	32	CRB2 ^2,3,6,7^	3(22): 0(0)	54	KAT5 ^3^	3(48): 1(5)
11	ITPR1 ^3,4,7^	3(52): 0(0)	33	YEATS2	3(22): 0(0)	55	PTPRQ ^1^	3(40): 1(5)
12	OAS1 ^1^	3(51): 0(0)	34	CRYBG2 ^1^	3(21): 0(0)	56	TSHZ2 ^1^	3(36): 1(9)
13	UNC8 ^4^	3(51): 0(0)	35	CSMD1 ^1,2,3^	3(21): 0(0)	57	KIF13B ^3,4^	3(35): 1(9)
14	KLHL29 ^1,2,7^	3(50): 0(0)	36	DNMBP ^1^	3(21): 0(0)	58	RGS12 ^4^	3(33): 1(11)
15	DIAPH3 ^3^	3(49): 0(0)	37	WNT8A ^2,3,6^	3(21): 0(0)	59	REXO1	3(32): 1(3)
16	POTEB3 ^1^	3(43): 0(0)	38	LILRA2 ^2^	3(20): 0(0)	60	CASZ1 ^2,3^	3(26): 1(8)
17	STAB2 ^2^	3(39): 0(0)	39	ADAMTS16	3(19): 0(0)	61	BCR^6^	3(25): 1(5)
18	CASP8AP2	3(32): 0(0)	40	IGLL1	3(19): 0(0)	62	LRP6 ^1,2,3,4,5,6^	3(24): 1(11)
19	GATAD2A ^3^	3(32): 0(0)	41	ITGB2 ^1^	3(19): 0(0)	63	DNAH12	3(22): 1(6)
20	GRN ^1,2,3,4,5,6,7^	3(32): 0(0)	42	PARP4 ^1^	3(19): 0(0)	64	ITGB1BP2 ^1^	3(11): 1(3)
21	TUBGCP6	3(31): 0(0)	43	CTCF ^1,2,3,7^	3(16): 0(0)			
22	THSD7B	3(30): 0(0)	44	IGSF1	3(16): 0(0)			

^1^—Alzheimer’s disease/dementia/amyloidosis (shaded); ^2^—inflammation/neuroinflammation; ^3^—neurogenesis; ^4^—ion channels; ^5^—brain injury/trauma/blood–brain barrier; ^6^—vasculature; ^7^—neuronal cell death.

## Data Availability

Data will be made available to qualified individual researchers on request if it does not conflict with IRB or institutional limitations.

## Supplementary Materials

The following are available online at https://www.mdpi.com/article/10.3390/brainsci11050583/s1, Table S1: LOOCV test metrics for randomized (RAND) group comparisons, Table S2: Mild AD > control, unfiltered, Table S3: Control > mild AD, unfiltered
