# Supplementary material for: Distinguishing Alzheimer’s Disease Patients and Biochemical Phenotype Analysis Using a Novel Serum Profiling Platform: Potential Involvement of the VWF/ADAMTS13 Axis
Source: Brain Sci. 2021 Apr 30;11(5):583. doi: 10.3390/brainsci11050583 (PMC8145311; doi:10.3390/brainsci11050583)
Supplement: Supplementary file 1 [file brainsci-11-00583-s001.zip › Supplement S1_hanas.pdf]

Supplement Table S1. LOOCV Test Metrics for randomized (RAND) group comparisons.

RAND: ESI-Ion Trap MS Group 1 vs. Group 2	Group 1 Mean (SD)	Group 2 Mean (SD)	*"effect size"*	*p*-value	N Group 1 : Group 2	Sensitivity	Specificity	Member of both FP & FN	Figure #
RAND: moderate-Alzheimer's Disease vs. RAND: Control, N=14	53.38% (7.75%)	47.20% (7.02%)	0.84	0.015	16 : 14	0.53	0.47	29	2D
RND: mild-Alzheimer's Disease vs. RAND: Control, N=14	47.03% (4.48%)	49.00% (3.52%)	0.48	0.09	15 : 14	0.52	0.48	29	2B
RAND: mild-Alzheimer's Disease vs. RAND: moderate-Alzheimer's Disease	56.53% (4.79%)	58.23% (5.52%)	0.32	0.183	15 : 16	0.69	0.4	31	3D
RAND: Alzheimer's Disease vs. RAND: Control, N=14	42.09% (5.23%)	39.29% (4.07%)	0.59	0.18	31 : 14	0.48	0.52	45	3B
RAND: Training set AD vs RAND: Control	46.38% (5.80%)	45.94% (5.90%)	0.07	0.43	16 : 14	0.53	0.48	29	3B
RAND: Mild and moderate-Alzheimer's disease vs RAND: TBI	49.68% (4.21%)	46.55 (4.69%)	0.70	0.034	18: 13	0.58	0.42	31	4C
RAND: mild-Alzheimer's Disease vs RAND: moderate-Alzheimer's Disease	56.16% (4.79%)	58.17% (5.71%)	0.38	0.14	15 : 16	0.52	0.52	31	4D
**RAND: ESI-single Quad MS Group 1 vs. Group 2**	**Mean (SD) Group 1**	**Mean (SD) Group 2**	***"effect size"***	***p*-value**	**N Group 1 : Group 2**	**Sensitivity**	**Specificity**	**Member of both FP & FN**	**Figure #**
RAND: mild-Alzheimer's Disease vs. RAND: Control	62.31% (12.07%)	51.19% (16.31%)	0.77	0.024	15 : 14	0.54	0.92	22	5A
RAND: moderate-Alzheimer's Disease vs. RAND: Control	53.88% (13.20%)	47.81% (7.62%)	0.56	0.065	16 : 14	0.79	0.32	18	5b
RAND: Alzheimer's Disease vs. RAND: Control	38.09% (7.13%)	29.83% (11.48%)	0.86	0.011	31 : 14	0.92	0.45	25	5C
RAND: moderate-Alzheimer's Disease vs. RAND: mild-Alzheimer's Disease	46.38% (5.93%)	40.38% (12.57%)	0.61	0.054	15 : 16	0.52	0.36	23	5D

Leave One Out Cross Validation (LOOCV); Mass Spectrometer (MS); Standard Deviation (SD); effect size measured by Cohen's *d*; p-value measured by Student's t-test; True Negative (TN); False Negative (FN);
