# Supplementary material for: Distinguishing Alzheimer’s Disease Patients and Biochemical Phenotype Analysis Using a Novel Serum Profiling Platform: Potential Involvement of the VWF/ADAMTS13 Axis
Source: Brain Sci. 2021 Apr 30;11(5):583. doi: 10.3390/brainsci11050583 (PMC8145311; doi:10.3390/brainsci11050583)
Supplement: Supplementary file 1 [file brainsci-11-00583-s001.zip › Supplement S2_hanas.pdf]

Supplement Table S2: Mild AD > Control, Unfiltered.

	Symbol	Mild: Control [#Sera(#Hits)]		Symbol	Mild: Control [#Sera(#Hits)]		Symbol	Mild: Control [#Sera(#Hits)]
1	SSPO	8(162): 4(57)	47	KMT2E	3(37): 0(0)	93	FREM2	3(24): 1(7)
2	IGK	8(221): 7(61)	48	SRRM2	3(37): 0(0)	94	KMT2C	3(24): 1(11)
3	MUC16	8(122): 7(273)	49	CNOT1	3(35): 0(0)	95	MT-ND6	3(22): 1(8)
4	IGH	8(318): 8(90)	50	POU4F3	3(35): 0(0)	96	PDZD2	3(22): 1(9)
5	TRB	7(53): 7(71)	51	CYLD	3(33): 0(0)	97	POLR2A	3(21): 1(60)
6	IGL	7(168): 8(118)	52	CAPN3	3(32): 0(0)	98	ADAMTS6	3(19): 1(3)
7	VWF	6(158): 1(15)	53	ITGAX	3(32): 0(0)	99	MALRD1	3(17): 1(8)
8	TRA	6(42): 4(27)	54	SMCHD1	3(30): 0(0)	100	PCDHB7	3(16): 1(7)
9	TTN	6(169): 5(76)	55	GIT1	3(29): 0(0)	101	SPEN	3(15): 1(5)
10	IGKC	5(130): 1(3)	56	ACACB	3(27): 0(0)	102	MYCBP2	3(15): 1(6)
11	IGLC2	5(83): 3(209)	57	C5orf42	3(26): 0(0)	103	HLA-DQA1	3(14): 1(5)
12	MUC5AC	5(153): 4(94)	58	KHSRP	3(26): 0(0)	104	TRIP11	3(13): 1(5)
13	F8	4(84): 0(0)	59	OR7G1	3(26): 0(0)	105	POLA1	3(9): 1(3)
14	CR1	4(30): 0(0)	60	ASCC1	3(25): 0(0)	106	MUC3B	3(9): 1(15)
15	UPF2	4(30): 0(0)	61	MFRP	3(25): 0(0)	107	CDCA2	3(8): 1(3)
16	LTBP2	4(66): 1(5)	62	SSC5D	3(25): 0(0)	108	SRCAP	3(54): 2(7)
17	ADGRV1	4(66): 1(15)	63	HNRNPA3	3(23): 0(0)	109	PKHD1	3(44): 2(19)
18	SVEP1	4(56): 1(7)	64	RXFP3	3(23): 0(0)	110	PEAR1	3(39): 2(6)
19	LRP4	4(42): 1(5)	65	AGAP2	3(22): 0(0)	111	MCPH1	3(37): 2(58)
20	ZNF469	4(37): 1(5)	66	NAAA	3(22): 0(0)	112	FRYL	3(33): 2(28)
21	FBN1	4(28): 1(14)	67	MEGF11	3(21): 0(0)	113	ZNF462	3(30): 2(18)
22	MT1B	4(17): 1(3)	68	ATMIN	3(19): 0(0)	114	ACTR3B	3(29): 2(11)
23	MT-ND1	4(127): 2(25)	69	EGR1	3(18): 0(0)	115	FCGBP	3(28): 2(9)
24	OTOGL	4(74): 2(25)	70	GOLGA2P11	3(16): 0(0)	116	USP34	3(24): 2(12)
25	NOTCH1	4(39): 2(22)	71	LOC400682	3(16): 0(0)	117	PRAG1	3(21): 2(20)
26	LRP1	4(34): 2(13)	72	TENM1	3(15): 0(0)	118	FLNC	3(20): 2(10)
27	MUC3A	4(26): 3(23)	73	PCDHA4	3(13): 0(0)	119	C2CD3	3(20): 2(14)
28	VWDE	4(22): 3(30)	74	TNFAIP3	3(13): 0(0)	120	ZFYVE9	3(18): 2(12)
29	HERC2	4(40): 4(30)	75	ZC3H4	3(12): 0(0)	121	TENM2	3(17): 2(23)
30	LRP2	4(19): 4(31)	76	FAM114A1	3(11): 0(0)	122	HERC1	3(16): 2(6)
31	NOTCH2	4(33): 5(34)	77	ARIH1	3(9): 0(0)	123	LOC101928841	3(15): 2(6)
32	DLGAP5	3(115): 0(0)	78	OIT3	3(8): 0(0)	124	FAM208B	3(15): 2(14)
33	BSG	3(72): 0(0)	79	IGLC3	3(259): 1(2)	125	IGLC1	3(230): 3(201)
34	NFXL1	3(59): 0(0)	80	CHFR	3(169): 1(22)	126	TNC	3(54): 3(38)
35	KIAA1109	3(55): 0(0)	81	POGZ	3(126): 1(26)	127	ADAM15	3(54): 3(72)
36	TNRC6A	3(53): 0(0)	82	AGAP6	3(119): 1(3)	128	HLA-A	3(41): 3(132)
37	ATP1A1	3(52): 0(0)	83	LTBP1	3(118): 1(25)	129	MUC4	3(12): 3(73)
38	SPINK5	3(51): 0(0)	84	HSPG2	3(73): 1(3)	130	MUC17	3(9): 3(20)
39	PCDH8	3(46): 0(0)	85	PRUNE2	3(59): 1(7)	131	FRAS1	3(60): 4(52)
40	ARHGEF2	3(45): 0(0)	86	SEMA4D	3(39): 1(36)	132	MYO7A	3(41): 4(64)
41	CFAP221	3(45): 0(0)	87	FRMPD1	3(32): 1(9)	133	FBN2	3(30): 4(86)
42	EYA1	3(45): 0(0)	88	RYR3	3(32): 1(15)	134	LAMA1	3(29): 4(51)
43	SARDH	3(44): 0(0)	89	MIDN	3(27): 1(6)	135	MUC2	3(13): 4(57)
44	RALGAPA2	3(41): 0(0)	90	CELSR2	3(25): 1(4)	136	MUC19	3(32): 5(38)
45	STXBP2	3(40): 0(0)	91	SEC24C	3(25): 1(5)	137	MUC5B	3(26): 5(63)
46	PKD1P6	3(39): 0(0)	92	NBPF10	3(24): 1(3)			
