# Supplementary material for: Distinguishing Alzheimer’s Disease Patients and Biochemical Phenotype Analysis Using a Novel Serum Profiling Platform: Potential Involvement of the VWF/ADAMTS13 Axis
Source: Brain Sci. 2021 Apr 30;11(5):583. doi: 10.3390/brainsci11050583 (PMC8145311; doi:10.3390/brainsci11050583)
Supplement: Supplementary file 1 [file brainsci-11-00583-s001.zip › Supplement S3_hanas.pdf]

Supplement Table S3: Control > Mild AD, Unfiltered.

	Symbol	Control: Mild [#Sera(#Hits)]		Symbol	Control: Mild [#Sera(#Hits)]		Symbol	Control: Mild [#Sera(#Hits)]
1	IGL	8(118): 7(168)	38	GATAD2A	3(32): 0(0)	75	TSHZ2	3(36): 1(9)
2	IGH	8(90): 8(318)	39	GRN	3(32): 0(0)	76	KIF13B	3(35): 1(9)
3	TRB	7(71): 7(53)	40	TUBGCP6	3(31): 0(0)	77	RGS12	3(33): 1(11)
4	MUC16	7(273): 8(122)	41	THSD7B	3(30): 0(0)	78	REXO1	3(32): 1(3)
5	IGK	7(61): 8(221)	42	ADAMTSL3	3(29): 0(0)	79	DNAH1	3(28): 1(33)
6	RIF1	5(105): 2(43)	43	AMBP	3(28): 0(0)	80	CASZ1	3(26): 1(8)
7	MUC5B	5(63): 3(26)	44	PDE9A	3(27): 0(0)	81	BCR	3(25): 1(5)
8	MUC19	5(38): 3(32)	45	SH3PXD2A	3(27): 0(0)	82	LRP6	3(24): 1(11)
9	NOTCH2	5(34): 4(33)	46	STK36	3(27): 0(0)	83	DNAH12	3(22): 1(6)
10	TTN	5(76): 6(169)	47	STXBP5L	3(26): 0(0)	84	PCDH17	3(13): 1(11)
11	ZNF142	4(67): 0(0)	48	PLXNB2	3(25): 0(0)	85	ITGB1BP2	3(11): 1(3)
12	BAZ1A	4(39): 0(0)	49	ZNF592	3(23): 0(0)	86	HIVEP1	3(85): 2(43)
13	LAMA3	4(46): 1(2)	50	CLDN7	3(22): 0(0)	87	CELSR1	3(48): 2(16)
14	NAV2	4(70): 2(34)	51	CRB2	3(22): 0(0)	88	NOTCH4	3(46): 2(21)
15	OBSCN	4(32): 2(17)	52	YEATS2	3(22): 0(0)	89	CRB1	3(33): 2(6)
16	MT-CO1	4(22): 2(9)	53	CRYBG2	3(21): 0(0)	90	FBN3	3(33): 2(29)
17	LRP1B	4(17): 2(6)	54	CSMD1	3(21): 0(0)	91	NEB	3(33): 2(154)
18	FBN2	4(86): 3(30)	55	DNMBP	3(21): 0(0)	92	CSMD3	3(30): 2(34)
19	MYO7A	4(64): 3(41)	56	WNT8A	3(21): 0(0)	93	CCDC168	3(29): 2(18)
20	MUC2	4(57): 3(13)	57	LILRA2	3(20): 0(0)	94	LRRK1	3(27): 2(10)
21	FRAS1	4(52): 3(60)	58	ADAMTS16	3(19): 0(0)	95	TRPM2	3(22): 2(14)
22	LAMA1	4(51): 3(29)	59	IGLL1	3(19): 0(0)	96	VWCE	3(19): 2(13)
23	LRP2	4(31): 4(19)	60	ITGB2	3(19): 0(0)	97	TECTA	3(18): 2(28)
24	HERC2	4(30): 4(40)	61	PARP4	3(19): 0(0)	98	STARD9	3(16): 2(10)
25	MUC5AC	4(94): 5(153)	62	CTCF	3(16): 0(0)	99	SIVA1	3(15): 2(14)
26	TRA	4(27): 6(42)	63	IGSF10	3(16): 0(0)	100	ARHGEF5	3(12): 2(8)
27	SSPO	4(57): 8(162)	64	BOC	3(14): 0(0)	101	MT-ND5	3(8): 2(6)
28	PITRM1	3(94): 0(0)	65	LRRC4	3(14): 0(0)	102	IGLC1	3(201): 3(230)
29	PCSK5	3(56): 0(0)	66	SNTB2	3(11): 0(0)	103	HLA-A	3(132): 3(41)
30	ITPR1	3(52): 0(0)	67	MC1R	3(10): 0(0)	104	MUC4	3(73): 3(12)
31	OAS1	3(51): 0(0)	68	RAB3GAP2	3(9): 0(0)	105	ADAM15	3(72): 3(54)
32	UNC80	3(51): 0(0)	69	POTEB	3(3): 0(0)	106	TNC	3(38): 3(54)
33	KLHL29	3(50): 0(0)	70	CYP4F11	3(143): 1(8)	107	MUC17	3(20): 3(9)
34	DIAPH3	3(49): 0(0)	71	KMT2A	3(124): 1(9)	108	VWDE	3(30): 4(22)
35	POTEB3	3(43): 0(0)	72	ADAMTS7	3(50): 1(18)	109	MUC3A	3(23): 4(26)
36	STAB2	3(39): 0(0)	73	KAT5	3(48): 1(5)	110	IGLC2	3(209): 5(83)
37	CASP8AP2	3(32): 0(0)	74	PTPRQ	3(40): 1(5)	111		
